# Study of Dimorphism Transition Mechanism of *Tremella fuciformis* Based on Comparative Proteomics

**DOI:** 10.3390/jof8030242

**Published:** 2022-02-28

**Authors:** Yaxing Li, Haohao Tang, Weichao Zhao, Yang Yang, Xiaolu Fan, Guanping Zhan, Jiahuan Li, Shujing Sun

**Affiliations:** 1 College of Life Science, Fujian Agriculture and Forestry University, Fuzhou 350002, China; lyx@fafu.edu.cn (Y.L.); 3200537101@fafu.edu.cn (W.Z.); 1210514107@fafu.edu.cn (Y.Y.); 1200514085@fafu.edu.cn (G.Z.); lijiahuansw05@fafu.edu.cn (J.L.); 2 Basic Forestry and Proteomics Research Center, Fujian Agriculture and Forestry University, Fuzhou 350002, China; tanghaohao0987@fafu.edu.cn (H.T.); 0000pct066@fafu.edu.cn (X.F.)

**Keywords:** proteomics, data-independent acquisition (DIA), parallel reaction monitoring (PRM), MAPK signaling pathway, metabolism

## Abstract

*Tremella fuciformis* is a dimorphic fungus that can undertake a reversible transition between yeast-like conidia and hyphal forms. The transformation mechanism and proteomic differences between these two forms have not been reported. Therefore, in this study, we attempted to explore the differential protein profiles of dikaryotic yeast-like conidia from fruiting bodies and mycelia (FBMds) and dikaryotic mycelia (DM) by synthetically applying high-resolution MS1-based quantitative data-independent acquisition (HRMS1-DIA) full proteomics and parallel reaction monitoring (PRM) targeted proteomics. The results showed that a total of 5687 proteins were quantified, and 2220 of them (39.01%) showed more than a two-fold change in expression. The functional analysis of the differentially expressed proteins (DEPs) confirmed that the DEPs were mainly located in the membrane and nucleus. The FBMds tended to express proteins involved in biosynthesis, metabolism, DNA replication and transcription, and DNA damage repair. At the same time, DM exhibited an increased expression of proteins involved in signal transduction mechanisms such as the mitogen-activated protein kinase (MAPK) signaling pathway and the Ras signaling pathway. Further, phosphorylation analysis confirmed the importance of the MAPK signaling pathway in *T. fuciformis* dimorphism, and comparative metabolism analysis demonstrated the metabolic difference between FBMds and DM. The information obtained in the present study will provide new insights into the difference between FBMds and DM and lay a foundation for further research on the dimorphism formation mechanism of *T. fuciformis*.

## 1. Introduction

*Tremella fuciformis* is a typical dimorphic fungus with two cell types in its life history, the yeast-like conidia form and the hyphal form, and it transforms under the influence of the environment [1,2]. The fruiting body of *T. fuciformis* is rich in nutrients and has high edible and medicinal value [3,4]. The lack of high-quality species and serious spawn degeneration are the main problems in the industrial production of *T. fuciformis*, which bring certain risks to breeding and production and cause huge economic losses. Therefore, finding robust isolates is very important for the industrial production of *T. fuciformis*. However, the dimorphism of *T. fuciformis* brings great difficulties to the breeding process, because it is very difficult to form mycelia [5] when basidiospores transform into yeast-like conidia. The dimorphism of *T. fuciformis* is an important theoretical basis for preserving, producing, cultivating, and breeding. At present, the reports on the dimorphism of *T. fuciformis* are mainly focused on the effect of environmental factors and phenotypic characteristics. Previous studies showed that the nitrogen source, carbon source, carbon/nitrogen ratio, pH value, temperature, culture time, and extracellular fluid of *Cinnamomum cinerea* all impacted the transformation from yeast-like conidia to hypha, and that multicarbon and multinitrogen sources and minerals such as phosphorus can also promote this transformation [6,7]. According to Zhu’s research, Tremella polysaccharide (TPS) from dikaryotic yeast-like conidia from fruiting bodies and mycelia (FBMds) and dikaryotic mycelia (DM) was mainly composed of xylose, mannose, glucose, and galactose. Still, the proportion of the sugar spectrum was different in two cell forms [8]. Some papers have also explored *T. fuciformis* dimorphism at the protein level. Isozyme electrophoresis showed differential expressions in esterase isozymes, polyphenol oxidase isozymes, and peroxidase isozymes produced by FBMds and DM [1]. TrGpa1 was also shown to be involved in the dimorphism of *T. fuciformis* and promote pseudohyphal growth [9]. There are few reports on the proteomics analysis of *T. fuciformis* dimorphism, and the dimorphism mechanism is still unknown. Notably, the dimorphism research involving other fungi has focused on proteogenomics and signaling pathways for several years. Researchers have found that many signaling pathways are involved in fungus dimorphism, including the mitogen-activated protein kinase (MAPK) signaling pathway, the cAMP–protein kinase A (cAMP–PKA) pathway, the target of rapamycin (TOR) pathway, the Rim101 pathway, and the Ca^2+^/calcineurin pathways [4,10,11,12,13]. Although much fruitful work has been carried out on fungal dimorphism, the signaling pathways in different fungi and the upstream and downstream components in the signaling pathways are still worth exploring. The expected results can be used as a reference for studying the dimorphism of *T. fuciformis*.

Differential proteomics has become a powerful tool for investigating cellular responses to various events and discovering biomarkers of biological processes. Over the past few decades, several data-dependent acquisition (DDA) based quantitative strategies such as 2D gel MS, isotope labeling, metabolic labeling, and label-free quantification have been applied for differential purposes. However, some drawbacks of these traditional approaches include quantifying differentially expressed proteins (DEPs), identifying low-abundance proteins, and isobaric interference [14,15,16]. Since the rise of sequential window acquisition of all theoretical mass spectra (SWATH-MS) as an emerging strategy with the advantages of an unbiased scan, high throughput, and high reproducibility, data-independent acquisition (DIA) has become a popular quantitative proteomics method applied to personalized medicine, biomarker research, drug screens, genetic association studies, and systems biology [17]. As a new strategy of DIA, high-resolution MS1-based quantitative data-independent acquisition (HRMS1-DIA) was developed from the traditional DIA method in the past two years. Compared with the traditional DIA method based on production quantification, HRMS1-DIA significantly improved the quantity and accuracy of protein quantification through its use of ultra-high-resolution primary full scan and MS1 quantification [18].

To explore the differential expression of proteins and find the potential underlying mechanism of *T. fuciformis* dimorphism, HRMS1-DIA-based proteomics technology was used to analyze the total proteins of FBMds and DM, followed by the bioinformatics analysis of DEPs. Parallel reaction monitoring (PRM) targeted proteomics and comparative metabolism were also used to confirm the predicted changes in the bioinformatics analysis. The results of our study will provide an omic insight into the dimorphism of *T. fuciformis*.

## 2. Materials and Methods

### 2.1. Fungus Strains and Culture Conditions

The wild-type strain *T. fuciformis* TWW01-AX was isolated from rotten wood (Anxi County, Quanzhou, China) by our laboratory and stored on agar slant at 4 °C. FBMds and DM were separately cultured on potato dextrose agar (PDA) solid medium covered with aseptic cellophane and cultivated in a constant-temperature incubator at 25 °C for 20 days. All reagents and chemicals were purchased from Sigma-Aldrich (St. Louis, MO, USA).

### 2.2. Protein Extraction, Digestion, and Peptide Fractions

Cell pellets were crushed into powder in liquid nitrogen, and 1 g powder was washed in 5 mL precooled TCA/acetone (10% trichloroacetic acid in acetone, precooled to −20 °C) twice. One milliliter of protein extraction buffer (2% volume of β-mercaptoethanol, 85% weight of phenol in ddH_2_O) was added to the powder, the extraction step was repeated 3 times, and the supernatant was combined. Subsequently, 5 times the volume of precooled methanol was added to the supernatant, and the mixture was precipitated overnight at −20 °C. The protein pellets were dissolved in lysis buffer (8 M urea, 100 mM Tris-HCl, pH 8.0, 1× protease inhibitor cocktail) and then measured by the BCA assay (ThermoFisher Scientific, Waltham, MA, USA). Protein digestion was performed with filter-aided sample preparation (FASP) method, as previously described in [19]. Briefly, lysates were loaded onto spin filter columns (Nanosep centrifugal devices with Omega membrane, 30 kDa MWCO; Pall, NY, USA) and reduced by DTT, followed by alkylation with iodoacetamide (IAA). Afterward, lysis buffer was exchanged by washing the membrane 3 times with 50 mM NH4HCO3. Proteins were digested overnight at 37 °C using trypsin (Promega, WI, USA) at an enzyme-to-protein ratio of 1:50 (*w*/*w*). Following the manufacturer’s protocol, peptide desalting was performed with the Pierce C18 spin tips (ThermoFisher Scientific, Waltham, MA, USA). Otherwise, the mixed peptides for the DDA library were preisolated to 10 fractions using high-pH reversed-phase HPLC (U3000 UHPLC System, ThermoFisher Scientific, Waltham, MA, USA), as previously described in [20]. Briefly, the peptide mixture was dissolving in 20 mM ammonium formate, loaded onto a reverse-phase column (Accucore C18 column, 2.1 mm × 150 mm, 1.9 μm; ThermoFisher Scientific, Waltham, MA, USA), separated, and collected under a 30 min linear gradient (from 5% ACN to 30% ACN, 20 mM ammonium formate, pH 10.0). The column flow rate was maintained at 0.3 mL/min, and the column temperature was maintained at 30 °C.

### 2.3. HRMS1-DIA-Based LC–MS/MS

DDA was performed to build the spectral library. Briefly, 10 peptide fractions were individually loaded onto the omics high-resolution series monolithic capillary HPLC columns (100 μM × 50 cm, KYOTO MONOTCHE) with a column temperature of 50 °C using the EASY-nLC1000 chromatographic system (ThermoFisher Scientific, Waltham, MA, USA) at a rate of 2.0 μL for 8 min. The peptides were subjected to a 120 min runtime elution at 600 nL/min using mobile phase A (0.1% formic acid in water) and phase B (0.1% formic acid in acetonitrile) with the following gradients: 0–4 min, 4–7% B, 4–79 min, 7–20% B, 79–108 min, 20–30% B, 108–110 min, 30–90% B, 110–120 min, and 90% B. The electrospray voltage of 2.2 kV versus the inlet of the Orbitrap Fusion Lumos (ThermoFisher Scientific, Waltham, MA, USA) was used and the mass spectrometry parameters were, briefly, as follows: (1) MS—scan range (*m*/*z*) = 350–1500, resolution = 60,000, AGC target = 4 × 10^5^, RF lens = 40%; (2) HCD-MS/MS—resolution = 30,000, AGC target = 5 × 10^4^, collision energy = 32, maximum injection time = 120 m, isolation window = 1.6 Da.

For HRMS1-DIA analysis, the chromatographic condition was set as the same as that of the DDA analysis, and the mass spectrometry parameters were set as previously described in [18], with some modifications. Briefly, the full MS experiment included one broadband scan acquired over *m*/*z* 350–1550 at a resolution of 120,000 with an AGC target value of 4 × 10^5^ and a maximum injection time of 50 ms. The MS/MS experiment included 20 scans/cycle (for a total of 60 scans) acquired at R =30,000 with an AGC target value of 2 × 10^5^, a maximum injection time of 72 ms, and HCD energy 32%.

### 2.4. Parallel Reaction Monitoring (PRM) Target Proteomics

No fewer than 3 unique peptides (unmodified, no missing cleavages) were selected as candidate proteins to perform PRM quantification. The chromatographic conditions were similar to those in the HRMS1-DIA experiment. The parameters of Orbitrap Fusion Lumos mass spectrometry were as follows: MS1 scan range was 400–1500 *m*/*z*, the resolution was 60 K, and AGC target was 4 × 10^5^; MS2 acquisition used the target MS2 module to monitor the target *m*/*z* list (Table S3) with a resolution of 30 K, isolation window 1.6 Da, AGC target 5 × 10^4^, maximum injection time 120 ms, HCD collision energy 35%, and retention time windows of 8 min around the expected precursor detection time.

### 2.5. Data Processing and Statistical Analysis

To obtain a confidential and comprehensive spectral library, DDA raw data and HRMS1-DIA raw data were both searched against the protein database by Spectronaut 15 (Biognosys AG, Switzerland) with default settings: carbamidomethyl (C) was fixed modification, oxidation (M) was variable modification, tolerance was 20 ppm, and precursor and protein false discovery rate (FDR) was 1%. Then, the HRMS1-DIA raw data underwent identification and quantification according to the following parameters: *q*-value cut-off applied for precursor and protein level was 1%, and decoy generation was set to mutate, which is similar to scrambled but only applies a random number of AA position swamps (min = 2, max = length/2). All selected precursors passing the filters were used for MS1 quantification. Interference peaks in the MS2 spectrum were removed, except for the three least-interfering peaks. The top 3 filtered peptides that passed the 1% *q*-value cut-off were used to calculate the major group quantities. The significance of log2-fold change values was determined using the Student’s one-tailed *t*-test (*p* < 0.05).

The PRM raw data were loaded into Protein Discoverer 2.2 (ThermoFisher Scientific, Waltham, MA, USA) to perform peptide identification, and the pdResult file containing peptide spectra was read by Skyline 20.1.0 [21]. Skyline 20.1.0 built the translation list and spectral library with a cut-off score >0.9; peptide length between 7 and 30 aa; and ion type b, y, and p; three productions with a *p*-value greater than 0.8 were used for peptide quantification and protein quantification.

### 2.6. Bioinformatics Analysis

Protein–protein interactions (PPIs) were analyzed by STRING online v11.5 [22] and Cytoscape v3.8.2 [23] against the homology species. The protein sequences were individually annotated by Blast2GO version 5 [24] and KOBAS 3 [25]. Then, the ClusterProfile package [26] was used to perform the gene ontology(GO) and Kyoto encyclopedia of genes and genomes (KEGG) pathway enrichment of 2-fold DEPs.

### 2.7. Metabolomics Analysis

Metabolite extraction was performed as previously described in [27]. Briefly, 50 mg freeze-dried cell pellets were added to 800 μL methanol. The mixture was ground with TissueLyser II (Qiagen, Dusseldorf, Germany) at 65 Hz for 90 s and kept at −20 °C for 1 h, then centrifuged at 12,000× *g* for 15 min. The supernatant was injected into a U3000 liquid chromatography system coupled to an Orbitrap Fusion system (ThermoFisher Scientific, Waltham, MA, USA) and an Accurose C18 column (150 mm × 0.21 mm × 1.9 μm, ThermoFisher Scientific, Waltham, MA, USA) to separate the derivatives under a 20 min gradient. Mass data were acquired under positive mode with the following parameters: full scan range 70–1000 *m*/*z;* 60 K mass resolution; dd-MS scan isolation window 1.6 Da; step collision energy 20%, 40%, 60%; 30 K mass resolution. Raw data were converted to MzXML and MGF files using Proteowizard software (version 3.0.6150), then Xcms software (version 1.46.0) was used for peak extraction and online MetDIA was used for metabolite identification and quantification [28,29].

## 3. Results

### 3.1. HRMS1-DIA Quantification of FBMds and DM

**Figure 1:**
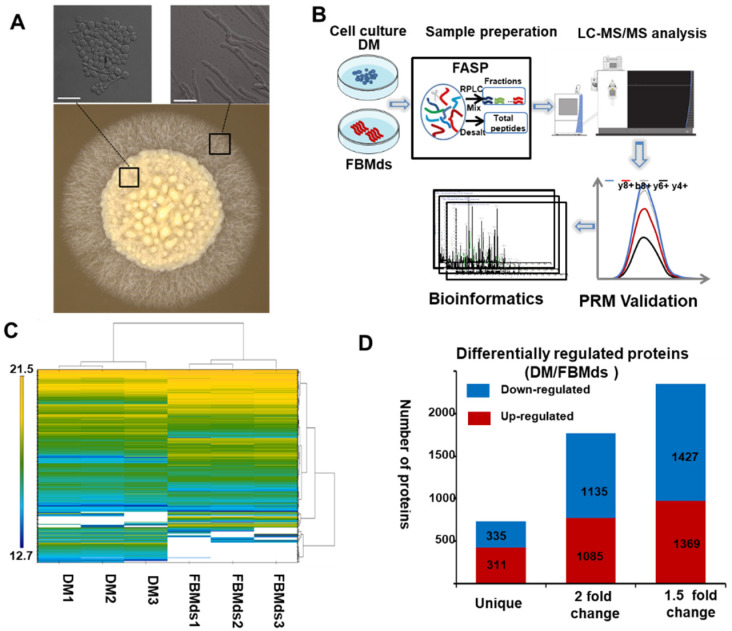
Proteomics data analysis of dikaryotic yeast-like conidia from fruiting bodies and mycelia (FBMds) and dikaryotic mycelia (DM) of *T. fuciformis* TWW01-AX. (**A**) Phenotypes and microscopic morphology were captured by stereo-scanning microscopy and a confocal microscope, respectively. Bar = 10 μm. (**B**) The flow chart of the proteomics analysis work. (**C**) Cluster analysis of protein expression intensity between three respective biological replicates of FBMds and DM: the color scale of log2 (intensity) is shown in the left, and white is a missing value. (**D**) Statics of differentially expressed proteins (DEPs) of DM/FBMds: red—the number of upregulated proteins in DM; blue—the number of downregulated proteins in DM.

A while after the FBMds had been inoculated on the germination medium, hyphae germinated around the colonies, and this phenomenon is called the dimorphism of *T. fuciformis* (Figure 1A). HRMS1-DIA and PRM were used to study the differential proteomics to understand the difference in protein expression between the two cell forms. The workflow chart is shown in Figure 1B.

In the HRMS1-DIA proteomic analysis, a total of 5687 proteins were quantified in three biological replicates with at least two matched unique peptides and FDR of 1% (Table S1). The cluster analysis of the protein expression intensity of *T. fuciformis* in the two cell forms is shown in Figure 1C, which illustrates the significant difference in protein expression between FBMds and DM. As Figure 1D shows, 311 proteins were specifically expressed in DM, and 335 proteins were expressed in FBMds; 2220 proteins (1135 downregulated and 1085 upregulated proteins, see Table S2) had more than a two-fold changed expression in DM.

In addition, both qualitative and quantitative repeatability of the experiments were observed. About 87% of the proteins identified in the triplicated experiments were involved in FBMds, and 93% in DM (Figure S1A,B). The correlation coefficients of the triplicated experiments were all greater than 0.9 (Figure S1C), indicating that quantitative information was obtained from the high-quality proteomics data.

### 3.2. Functional Analysis of DEPs of T. fuciformis Dimorphism

**Figure 2:**
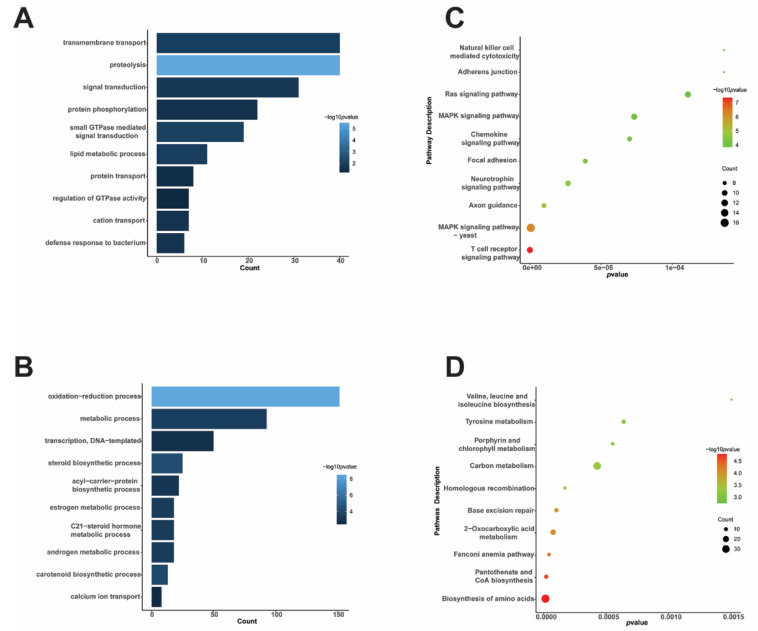
Visualization of top ten gene ontology (GO) terms of biological processes (BP) and Kyoto encyclopedia of genes and genomes (KEGG) enriched pathways. (**A**) GO terms of upregulated proteins and (**B**) GO terms of downregulated proteins in DM: the –log10 (*q*-value) color scale is shown on the right. (**C**,**D**) KEGG enriched pathways of upregulated proteins and downregulated proteins in DM, respectively: the *q*-value color scale and size scale of protein counts are shown on the right.

To better understand the biological characteristics of *T. fuciformis* dimorphism, GO enrichment analysis of the 2-fold DEPs was conducted using the ClusterProfile package in R studio v1.3. In the biological process (BP) classification, transmembrane transport, proteolysis, signal transduction, protein phosphorylation, and small-GTPase-mediated signal transduction were the five most enriched GO terms in upregulated proteins of DM. In contrast, several biological processes (oxidation–reduction, metabolism, and transcription) were the most enriched GO terms in downregulated proteins (Figure 2A,B). As shown in Figure S2, molecular function (MF) terms such as signal transducer activity, phosphotransferase activity, and GTP binding were upregulated in DM. In contrast, sequence-specific DNA binding, catalytic activity, oxidoreductase activity, and RNA polymerase II transcription factor activity were downregulated. Additionally, cellular component (CC) classification showed that the DEPs were significantly enriched in the membrane and nucleus (*p* < 0.05).

Furthermore, KEGG enrichment analysis of the DEPs showed that many signaling pathways had upregulated expression in DM, such as the MAPK signaling pathway (protein count = 16, *p*-adjust = 6.63 × 10^−7^), the Ras signaling pathway (protein count = 11, *p*-adjust = 1.11 × 10^−4^), the chemokine signaling pathway (protein count = 9, *p*-adjust = 7.60 × 10^−4^), and the neurotrophin signaling pathway (protein count = 10, *p*-adjust = 2.68 × 10^−5^). On the contrary, compared to FBMds, basic metabolism processes such as the biosynthesis of amino acids (protein count = 37, *p*-adjust = 1.34 × 10^−5^), carbon metabolism (protein count = 32, *p*-adjust = 4.28 × 10^−4^), pantothenate and CoA biosynthesis (protein count = 11, *p*-adjust = 2.01 × 10^−5^), and base excision repair (protein count = 11, *p*-adjust = 1.01 × 10^−4^) were downregulated. KEGG enrichment analysis showed that basic synthesis and metabolism activities of DM were lower than those of FBMds, but some signaling pathways were more active in DM (Figure 2C,D).

**Figure 3:**
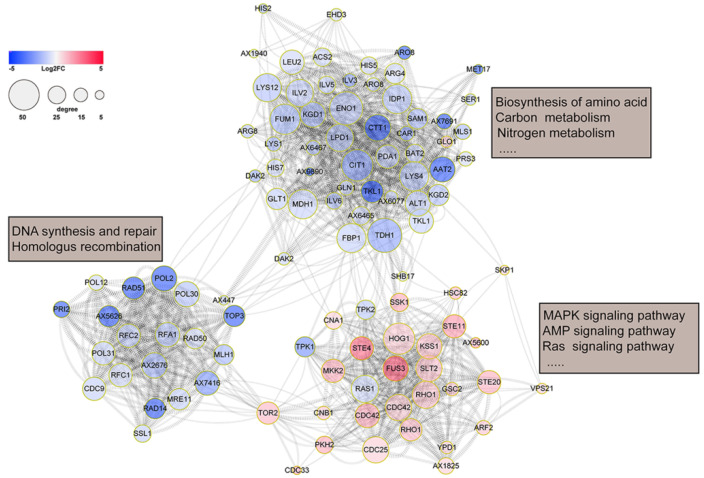
Visualization of significantly enriched KEGG pathways and predicted protein–protein interaction network of DEPs. The color scale of log2 (fold change) and the size scale of interaction edges are shown.

The STRING online and Cytoscape V3.8.3 software were used to further analyze the PPI network based on the KEGG enrichment pathways. Interestingly, the significantly enriched KEGG pathways formed a complex PPI network containing three subnetworks (Figure 3). The downregulated proteins mainly formed two PPI subnetworks by interacting with POL2, POL30, RAD51, KGD1, GLN1, and DM1. One subnetwork was related to biosynthesis and metabolism, the other was related to DNA synthesis and repair and homologous recombination. On the other hand, the upregulated proteins mainly forming signaling pathways also displayed a PPI subnetwork centering on the MAPK signaling pathway. It is noteworthy that many mitogen-activated protein kinases such as Hog1 (AX989), Slt2 (AX1207), Fus3 (AX1574), and STE4 (AX7569) were in the center of the network, acting as hub proteins.

### 3.3. PRM Validation of HRMS1-DIA Proteomics Results

**Figure 4:**
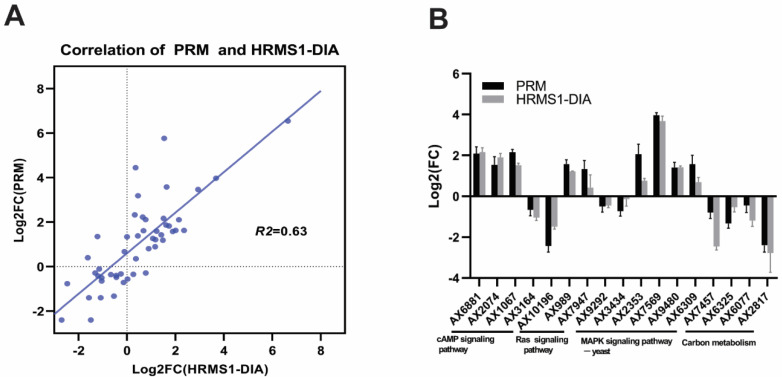
Parallel reaction monitoring (PRM) validation of high-resolution MS1-based quantitative data-independent acquisition (HRMS1-DIA) proteomics quantification. (**A**) Correlation between PRM quantification and HRMS1-DIA quantification of all 50 selected proteins. (**B**) The comparison of proteins related to significantly enriched KEGG pathways between PRM and HRMS1-DIA data.

To validate the reliability of the HRMS1-DIA results, 50 proteins (241 related peptides, Table S3) were selected based on the functional analysis to perform a PRM experiment. Of the 50 proteins, 44 exhibited a similar expression tendency, compared to the HRMS1-DIA results, except for AX9487, AX9163, AX9121, AX4761, and AX761 (Figure 4A, Table S4). The R-square of the PRM and HRMS1-DIA quantification ratio was 0.63. Furthermore, the validated proteins related to the MAPK signaling pathway, the Ras signaling pathway, the cAMP signaling pathway, and carbon metabolism were positively validated in this PRM experiment (Figure 4B), demonstrating that our proteomics data were considered reliable.

### 3.4. MAPK Signaling Pathway in T. fuciformis

**Figure 5:**
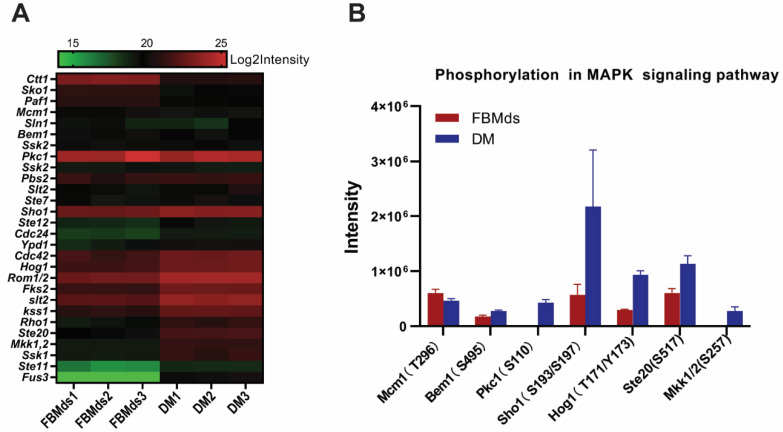
Protein expression and phosphorylation level of Mitogen-activated protein kinase (MAPK) signaling pathway. (**A**) Heatmap of proteins in MAPK signaling pathway between FBMds and DM: the protein names on the left are homologous with *Saccharomyces cerevisiae*. (**B**) Column chart of phosphorylation site intensity of MAPK signaling pathways in FBMds and DM, respectively.

As the most enriched pathway in DM, a total of 28 proteins assigned to the MAPK signaling pathway were quantified by proteomic data, more than half of which were upregulated, while only 3 proteins were downexpressed. As shown in Figure 5A, many mitogen-activated protein kinases such as Hog1, slt2, kss1, Ste20, Mkk1,2, Ste11, and Fus3 were upregulated in DM. In particular, four MAP kinases (Fus3, slt2, Hog1, and Kss1) directly interacting with the transcription factor showed immense changes in expression. Among the three downregulated proteins, two proteins (Paf1 and Sko1) belonged to the downstream transcription factors of the MAP kinase. Because the MAPK signaling pathway is a high-phosphorylation-level pathway, the phosphorylated proteins in the MAPK signaling pathway were further checked. As Figure 5B shows, five phosphorylation sites of four kinases (T171/Y173 of Hog1, S110 of Pkc1, S257 of Mkk1/2, and S517 of Ste20) were upregulated in DM. The spectrum of phosphorylation peptides is shown in Table S5. In summary, the differences in the proteins’ expression and the phosphorylation levels of the MAPK signaling pathway revealed that this pathway was more active in DM than FBMds.

### 3.5. Comparative Metabolism of FBMds and DM

**Figure 6:**
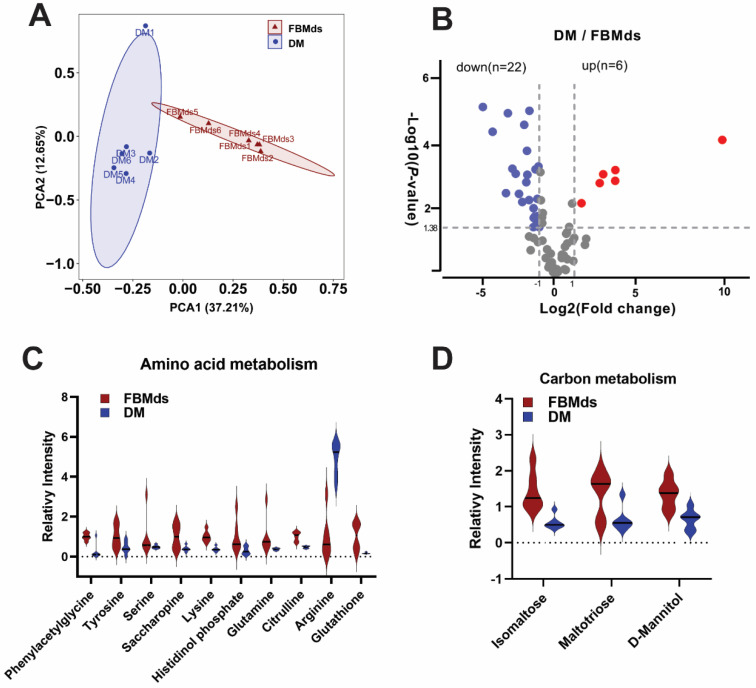
Comparative metabolism analysis of FBMds and DM. (**A**) Principal Components Analysis (PCA) plot of the metabolic signatures of FBMds and DM. (**B**) Volcano plot of the 75 known metabolites used for analyses. (**C**,**D**) Comparison of metabolites related to amino acids and carbohydrates between FBMds and DM, respectively.

Considering that metabolic processes such as carbon metabolism and the biosynthesis of amino acids differed between FBMds and DM in the proteomics analysis, six biological replicates per cell type were used to perform a comparative metabolism analysis using LC–MS/MS. Principal component analysis (PCA) confirmed the clear distinction between FBMds and DM, with about 50% of the variance explained by factors 1 and 2 (Figure 6A), and 22 downregulated metabolites and 6 upregulated metabolites in DM were identified with a 2-fold change and a *p* < 0.05 cut-off (Figure 6B). Among the different regulated metabolites, nine amino acids or intermediate products (phenylacetylglycine, tyrosine, serine, leucine, histidinol phosphate, histidinol phosphate, and citrulline) were downregulated, and only (arginine) was upregulated in DM. Additionally, three metabolites (isomaltose, maltotriose, and mannitol) related to carbohydrate digestion and absorption were downregulated (Figure 6C,D). The comparative metabolism analysis indicated that amino acid metabolism and carbon metabolism in DM are less active than in FBMds, which was consistent with the bioinformatics analysis of the HRMS1-DIA proteomics.

## 4. Discussion

There have been many reports on the proteome of dimorphic fungi, especially pathogenic fungi, but a proteomic analysis of *T. fuciformis* has not yet been reported. In this study, we attempted to analyze the dimorphism of *T. fuciformis* based on the protein database predicted by the genome sequence analysis of the wild-type strain *T. fuciformis* TWW01-AX. A total of 5687 proteins (55% of the protein database) and 38,965 peptides (about seven peptides per protein) were quantified with good repeatability, which offered a high coverage regarding both protein level and peptide level. Of the quantified proteins, 39% showed more than a two-fold changed expression, indicating that the proteomics profile of *T. fuciformis* undergoes a great change during the dimorphism process.

In this study, a large proportion of DEPs were quantified when yeast transformed into hyphae. Arginine plays a central role in the germination and growth of mycelial morphology in some dimorphous fungi. For example, the deletion of the ARG1 and ARG3 genes related to arginine synthesis can inhibit the transformation from yeast to mycelial cells. In *Zizania latifolia*, arginine promotes MT-type mycelial growth and inhibits the morphological transformation of T-type strains [30,31]. Under the action of arginase and urea hydrolase, CO_2_ produced by arginine metabolism can also promote the transformation of yeast into mycelial cells in *Candida albicans*. Still, when the encoding gene of urea hydrolase was knocked out, *Candida albicans* could not form germ tubes [32]. Interestingly, our proteomics and metabolism results found that arginine biosynthesis glutamyl dehydrogenase (AX4598), arginase (AX6366), acetylornithine transaminase (AX6129), arginosuccinase (AX6827), and the final product arginine were indeed upregulated to varying degrees in DM (Figure S3A). Superoxide dismutases (SODs) and thioredoxins (Trxs) are important for the mycelial phase to protect against oxidative stress, which is also the self-protection mechanism of pathogenic dimorphic fungi responding to adverse external stimulation when the host changes [33,34]. According to the proteomics data, SODs (AX4369 and AX10029) were downregulated, while Trxs (AX5890, AX8, AX3278, and AX4299) were unchanged or even upregulated (Figure S3B). As an adhesion factor for *T. marneffei* conidial attachment, glyceraldehyde-3-phosphate dehydrogenase (GAPDH) was upregulated in mycelia [35], but the intensity of GAPDH (AX3569) in DM was lower than that in FBMds. According to the proteomics data presented in this study, Lpd1 was identified in *C. albicans* as a hypha-specific protein [36], but the homologous protein of Lpd1 (AX1116) also had a higher expression in FBMds (Figure S3C,D). The above comparison shows the variations in dimorphic differential protein expression between different fungi; thus, the function of homologous proteins in different dimorphic fungi is still worth studying.

The bioinformatics results showed that several biological processes, such as the oxidation–reduction process; the metabolic process; transcription; and DNA synthesis, repair, and homologous recombination, were enriched in downregulated proteins in DM. Similar results were observed in other dimorphic fungi. Several proteins related to protein synthesis and transcription were upregulated in the conidia of *Aspergillus nidulans* [37]. Conidia from *A. nidulans* also keep an abundant reserve pool of mRNA and ribosomes before the fungus starts the germination process [38]. This indicates that metabolic activity allows for more flexibility when a fungus starts the germination process and explains why the increment rate of FBMds was higher than that of DM, with more damage-repair activity necessary for rapid growth.

Several signaling pathways, mainly including the MAPK signaling pathway, the cAMP–PKA pathway, the TOR pathway, the Rim101 pathway, and the Ca^2+^/calcineurin pathway, were reported to be related to fungal dimorphism. The MAPK signaling pathway is conserved in eukaryotic cells, amplifying extracellular signals through the step-by-step phosphorylation process so that cells can easily perceive changes in the external environment. In *Saccharomyces cerevisiae*, the MAPK signaling pathway mainly includes Fus3-mediated pheromone response, Kss1-mediated filamentation and invasive growth, Slt2-mediated cell wall integrity, and Hog-mediated high-osmolarity stress response [39,40]. The Fus3-MAPK signaling pathway plays an important role in the morphological transformation of dimorphic fungi. In *Saccharomyces cerevisiae*, Fus3, as the downstream primary protein kinase of the mating pheromone signal pathway, is mainly involved in the response to mating pheromones and cell fusion [41]. In *Ustilago maydis*, Kpp2, the homologous protein of Fus3, is very important for germ tube formation. When Kpp2 was knocked out, hyphae-formation ability and the perception of mating pheromones were greatly weakened, so the pathogenicity also was reduced [42,43]. Our data showed that the MAPK signaling pathway was highly upregulated in DM, with 16 upregulated proteins, and that the MAP kinases Fus3 (AX1574), Slt2 (AX1270), Kss1 (AX1067), and Hog (AX989) were all upregulated in DM, especially Hog (AX989), which leads to high-level phosphorylation. The cAMP–PKA signaling pathway also plays an important role in fungal dimorphism. In *Saccharomyces cerevisiae* and *Candida albicans*, extracellular signals are transmitted to small G protein Ras1/Ras2 and G protein α subunit Gpa1 through cell membrane receptor Gpr1/Mep2, thus activating adenylate cyclase Cyr1 to regulate the concentration of second messenger cAMP. Then, cAMP further activates PKA to phosphorylate downstream target proteins and promote mycelium growth [44,45]. KEGG enrichment also showed that this pathway was highly upregulated in hyphae. In *Schizosaccharomyces japonicas* and *Paracoccidioides brasiliensis*, Ras1–cdc42 and Ras-GTPase–Hog1 interaction regulated mycelial growth, and the Ras signaling pathway cooperated with the MAPK signaling pathway by the interactions of Ras1–cdc42 and Ras-GTPase–Hog1 [40,46]. In our results, it was interesting that the MAPK signaling pathway, the cAMP signaling pathway, and the Ras signaling pathway formed a complex regulatory network, including the activation of phosphorylated modifications. Therefore, we also saw the enrichment of phosphorylated molecules in the mycelial state, which corresponded to the phosphorylation of kinases and transcription factors in the MAPK signaling pathway of *T. fuciformis*. All these results showed that the dimorphism regulation of *T. fuciformis* is a complex network involving multiple signaling pathways. The MAPK signaling pathway may play the most important role in the network. Next, it is very important to verify the hub proteins of the MAPK signaling pathway affecting dimorphism in *T. fuciformis* and study its upstream and downstream interaction factors: MAP kinases and their direct-acting transcription factors seem to be good candidates.

## 5. Conclusions

This study used HRMS1-DIA-based and PRM targeted proteomics to compare the differential protein abundance between FBMds and DM of *T. fuciformis* TWW01-AX. The results revealed a large difference in protein levels between FBMds and DM, which involved many biological processes such as carbon metabolism and amino acid metabolism; the subsequent comparative metabolism analysis further demonstrated that the metabolic process was highly implicated in FBMds. Additionally, several signaling pathways such as the MAPK signaling pathway, the Ras signaling pathway, and the cAMP signaling pathway may regulate the morphological transformation of *T. fuciformis* by forming a complex network centering on the MAPK signaling pathway. The results of this study provide proteomic insights into *T. fuciformis* dimorphism.

## Data Availability

The mass spectrometry proteomics data were deposited in the ProteomeXchange consortium via the PRIDE partner repository with the dataset identifier PXD029989.

## Supplementary Materials

The following supporting information can be downloaded at: https://www.mdpi.com/article/10.3390/jof8030242/s1, The mass spectrometry proteomics data were deposited to the ProteomeXchange consortium via the PRIDE partner repository with the dataset identifier PXD029989. Figure S1: The repeatability of biological samples of proteomics, Figure S2: Visualization of top ten GO terms of molecular function (MF) and cell compounds (CC), Figure S3: Some protein expression levels in FBMds and DM, Table S1: Quantification information of HRMS1-DIA comparative proteomics, Table S2: The DEPs of DM vs. FBMds, Table S3: Candidate peptides for PRM validation, Table S4: PRM quantification results of candidate proteins, Table S5: Spectra of phosphopeptides in MAPK signaling pathway.

## Abbreviations

FBMds	Dikaryotic yeast-like conidia from fruiting bodies and mycelia
DM	Dikaryotic mycelia
DIA	Data-independent acquisition
HRMS1-DIA	high-resolution MS1-based quantitative data-independent acquisition
PRM	Parallel reaction monitoring
DEPs	Differentially expressed proteins
GO	Gene ontology
KEGG	Kyoto encyclopedia of genes and genomes
MAPK	Mitogen-activated protein kinase
TPS	Tremella polysaccharide
cAMP–PKA	cAMP–protein kinase A
TOR	Target of rapamycin
DDA	Data-dependent acquisition
SWATH-MS	Sequential window acquisition of all theoretical mass spectra
PDA	Potato dextrose agar
FDR	False discovery rate
BP	Biological process
MF	Molecular function
CC	Cellular component
PCA	Principal components analysis
