# Supplementary material for: Study of Dimorphism Transition Mechanism of Tremella fuciformis Based on Comparative Proteomics
Source: J Fungi (Basel). 2022 Feb 28;8(3):242. doi: 10.3390/jof8030242 (PMC8955754; doi:10.3390/jof8030242)
Supplement: Supplementary file 1 [file jof-08-00242-s001.zip › Figures S1-S3.pdf]

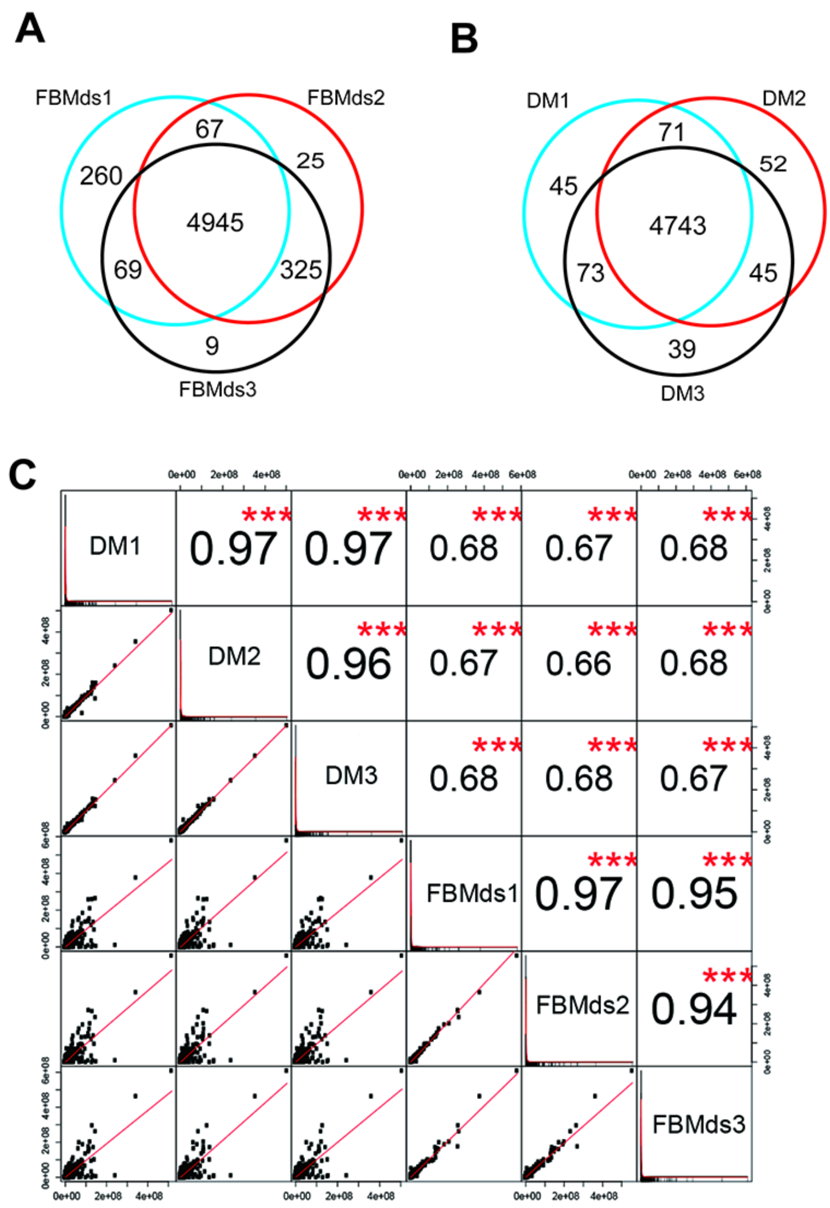

Figure S1. The repeatability of biological samples of proteomics. (A-B) The Venn diagram of protein identification numbers among three biological replicates in FBMds and DM, respectively. (C) The quantified protein intensity correlation (Log10 scale) among three FBMds and DM biological replicates. Pearson's p-value bar is shown in Tables.

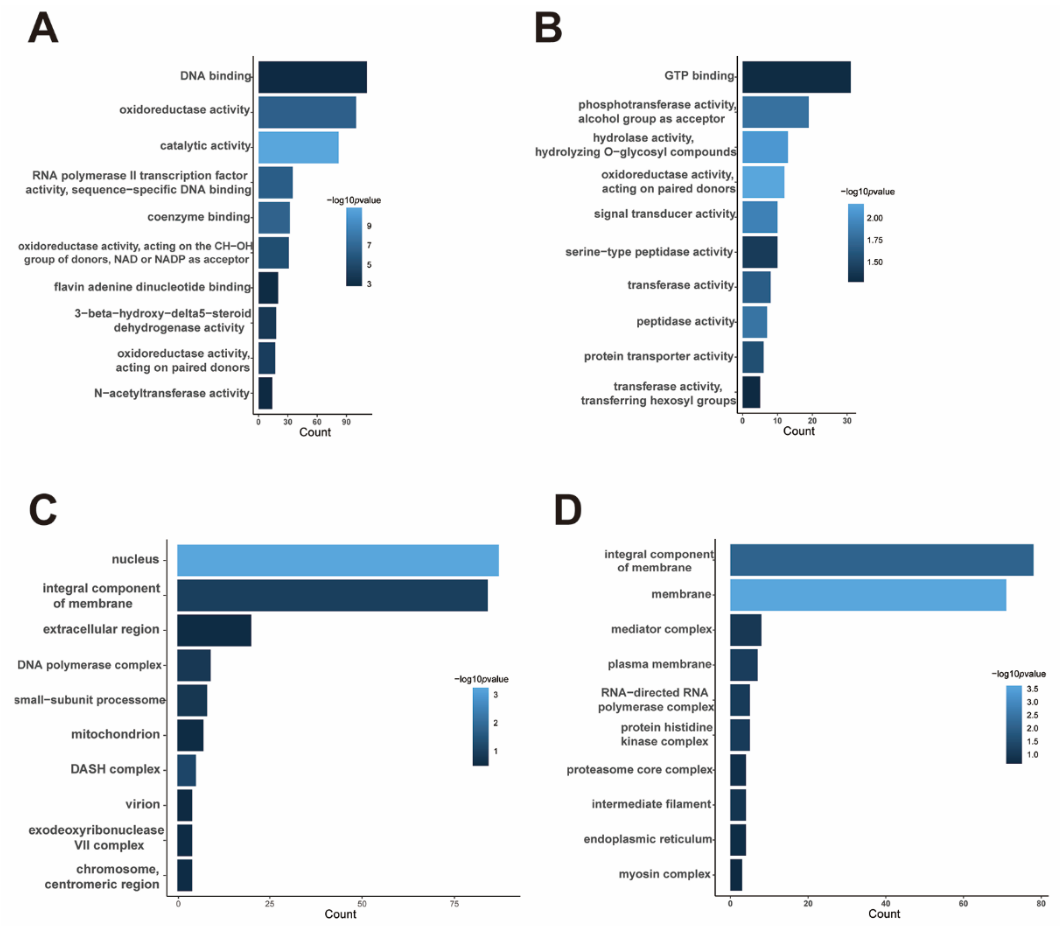

Figure S2. Visualization of top ten GO terms of molecular function (MF) and cellular compounds (CC). A and B: the down-regulated proteins enriched MF terms and the up-regulated MF terms, respectively. C-D: the down-regulated proteins enriched CC terms and the up-regulated CC terms, respectively.

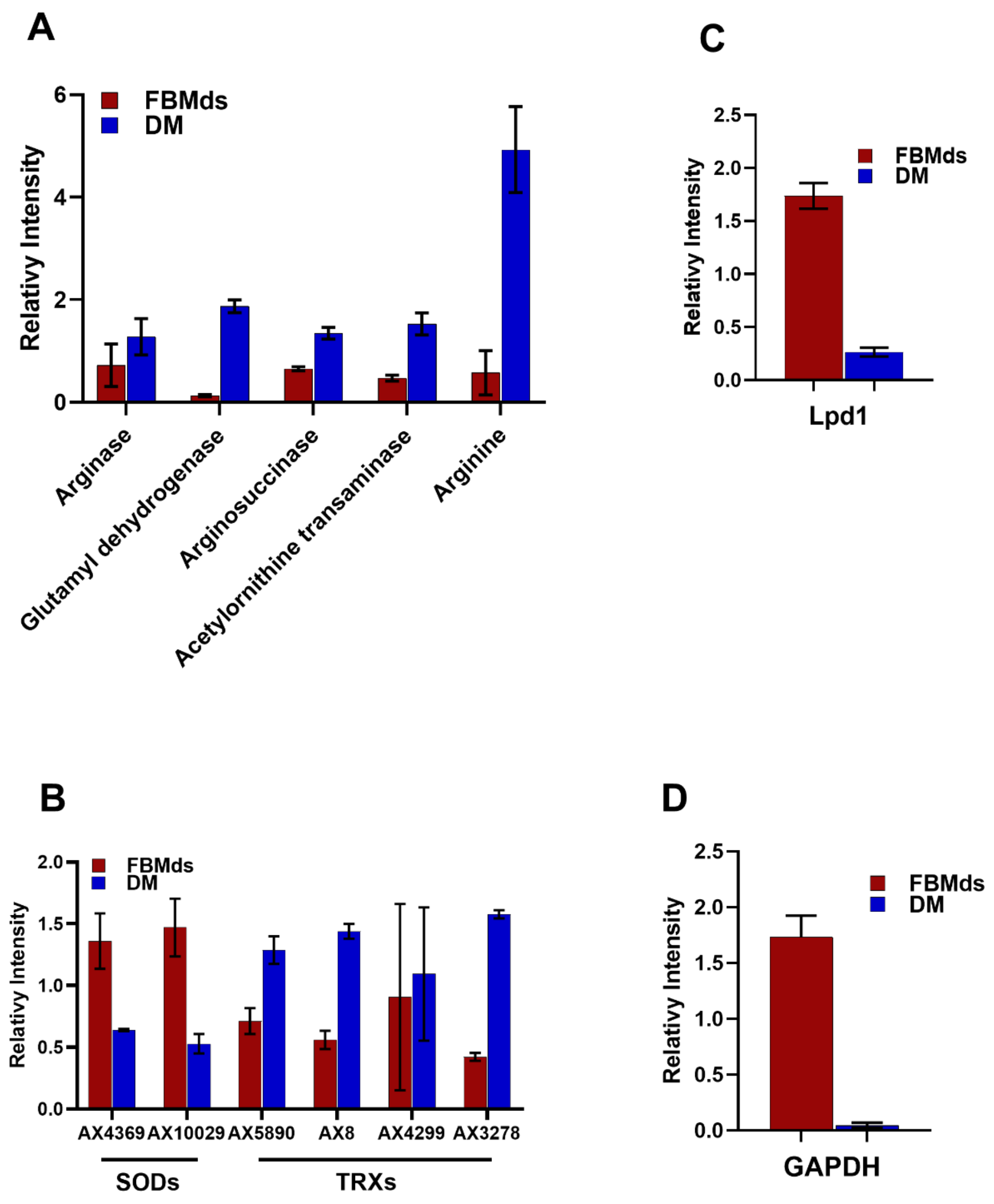

Figure S3. Some proteins expression levels in FBMds and DM.(A) Arginine and arginine related proteins comparative expression in FBMds and DM. (B). SODs and TRXs comparative expression in FBMds and DM. (C-D). Lpds and GAPDH comparative expression, respectively.
