# Supplementary material for: Study of Dimorphism Transition Mechanism of Tremella fuciformis Based on Comparative Proteomics
Source: J Fungi (Basel). 2022 Feb 28;8(3):242. doi: 10.3390/jof8030242 (PMC8955754; doi:10.3390/jof8030242)

Table S5 Spectrums of phospho-peptides in MAPK signaling pathway

1. **Evidence of tf*Bem*1-S495**

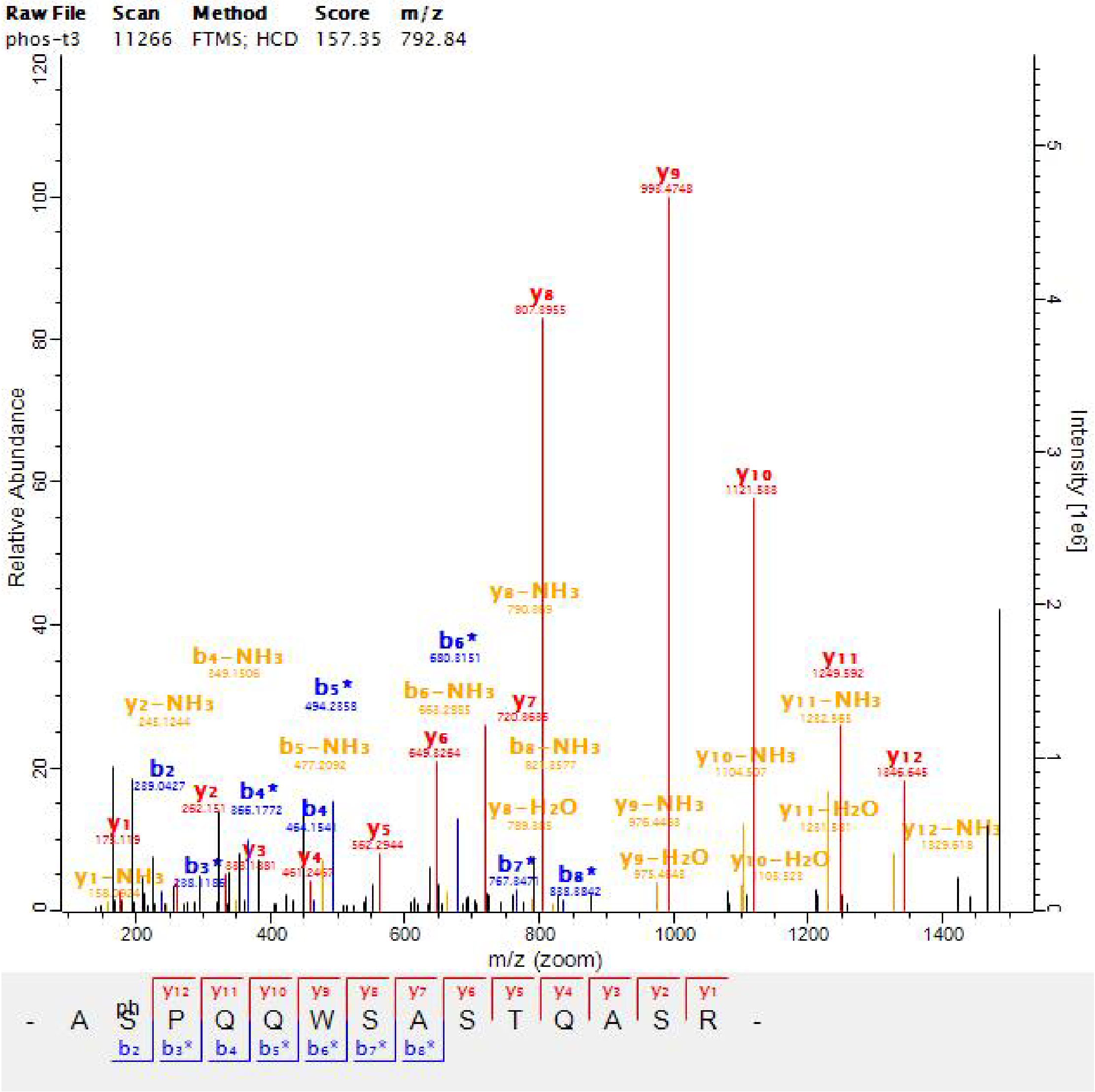

2. Evidence of tf*Hog*1-T171/Y173

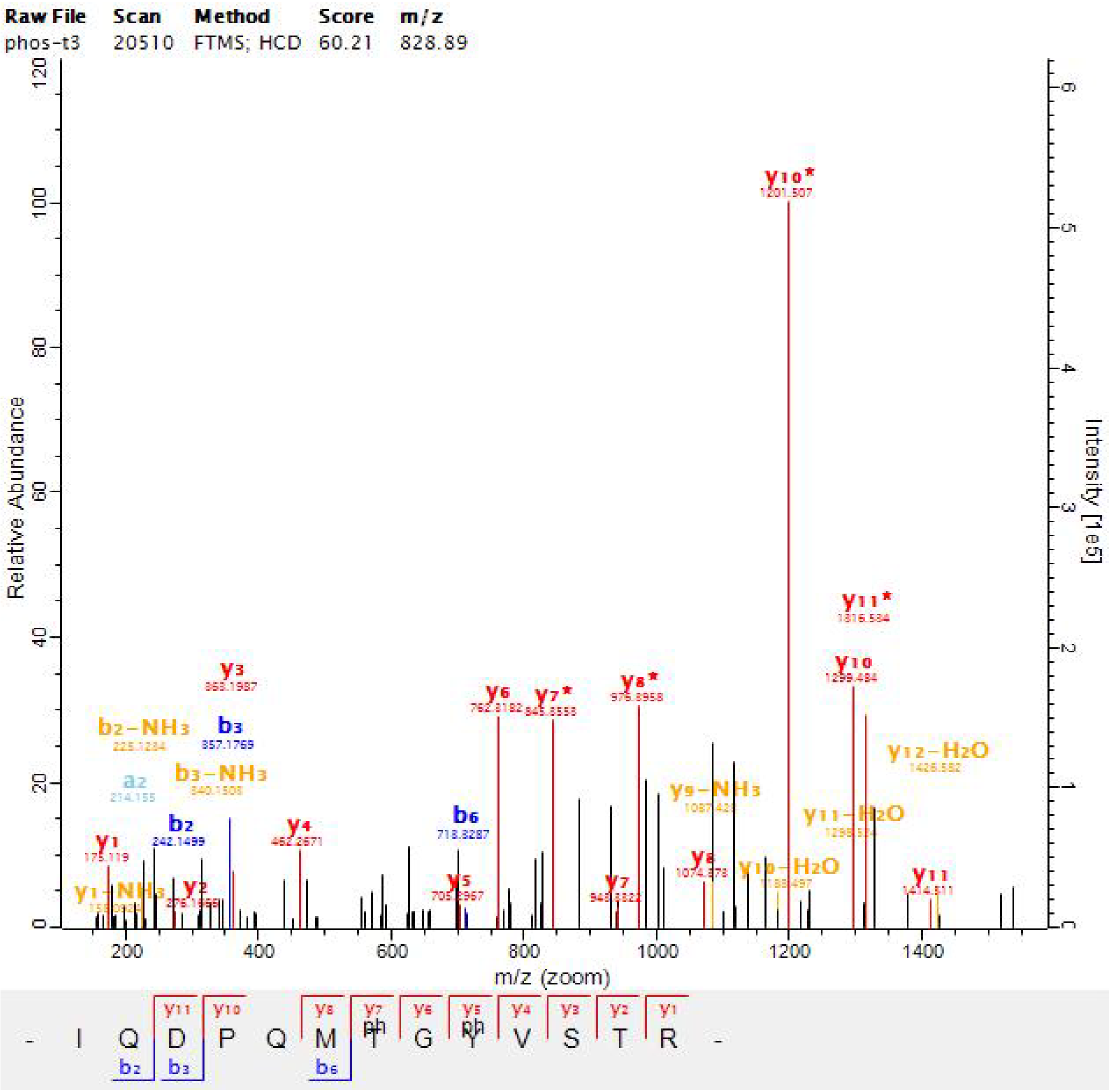

3. Evidence of tf*Mcm*1-T296

Raw File	Scan	Method	Score	m/z
phos-t1	47530	FTMS; HCD	93.55	823.09

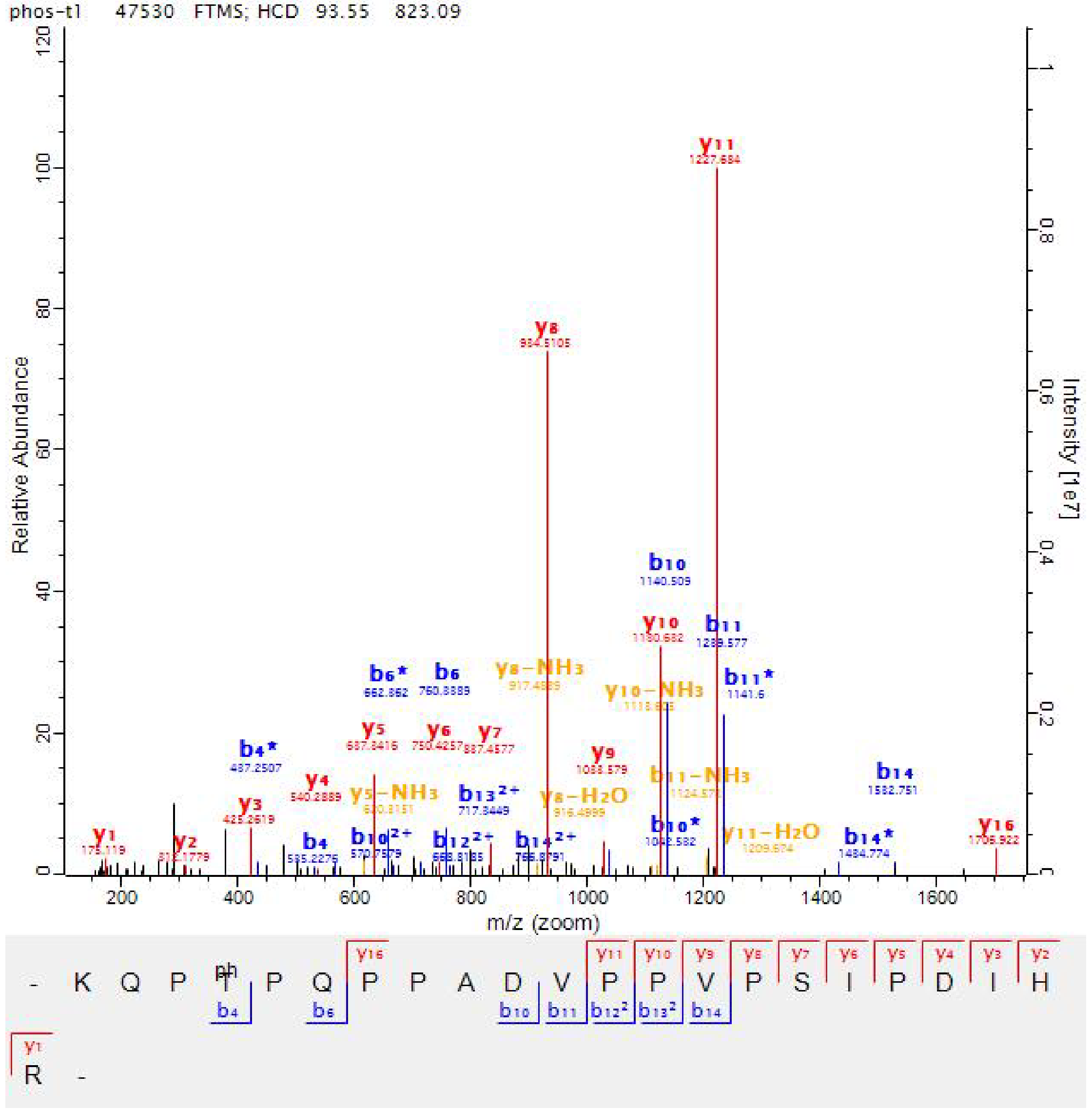

4. Evidence of tf*Mkk*1/2-S257

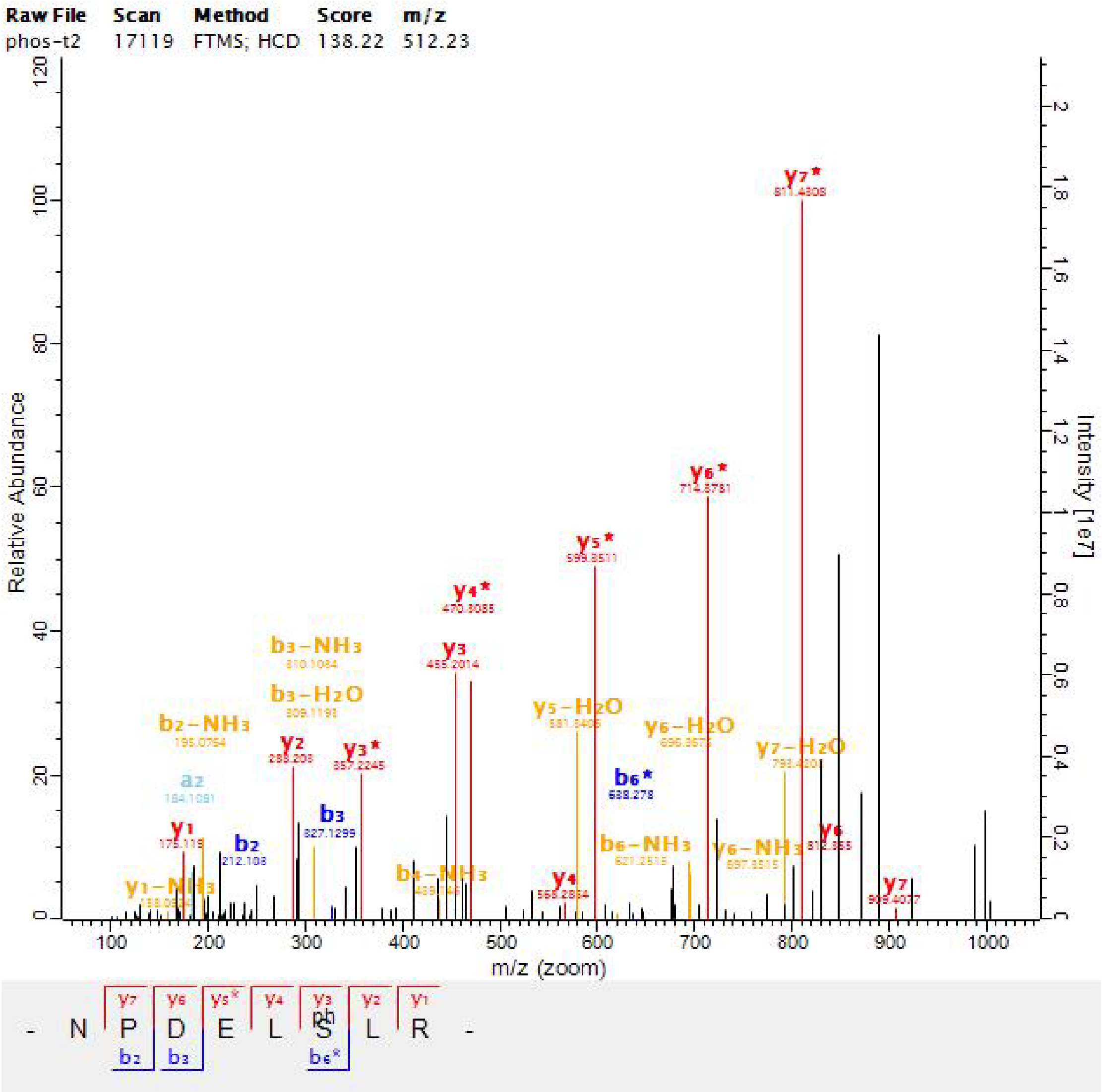

5. Evidence of tf*Pkc*1-S110

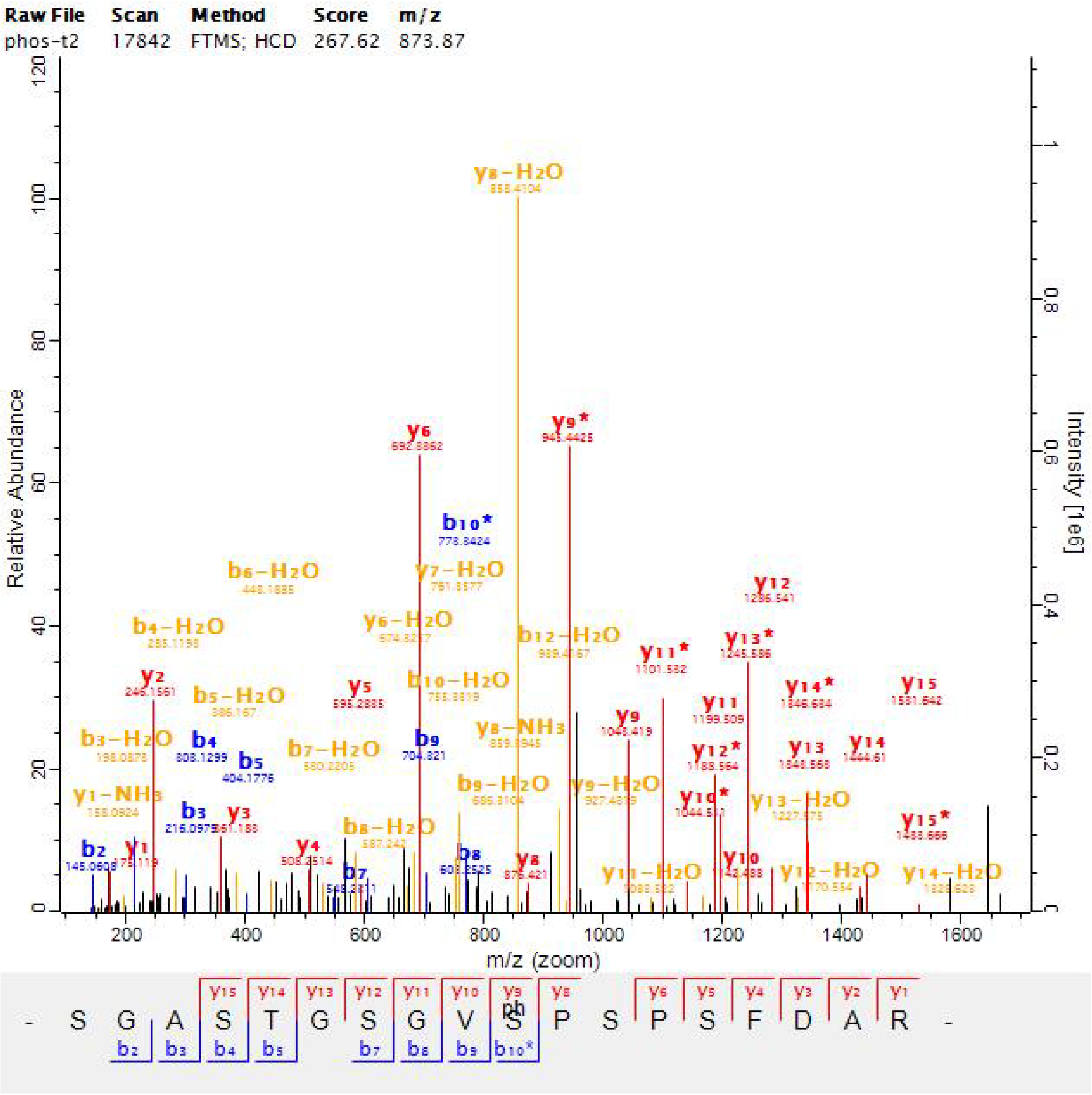

6. Evidence of tf*Sho*1-S193/S197

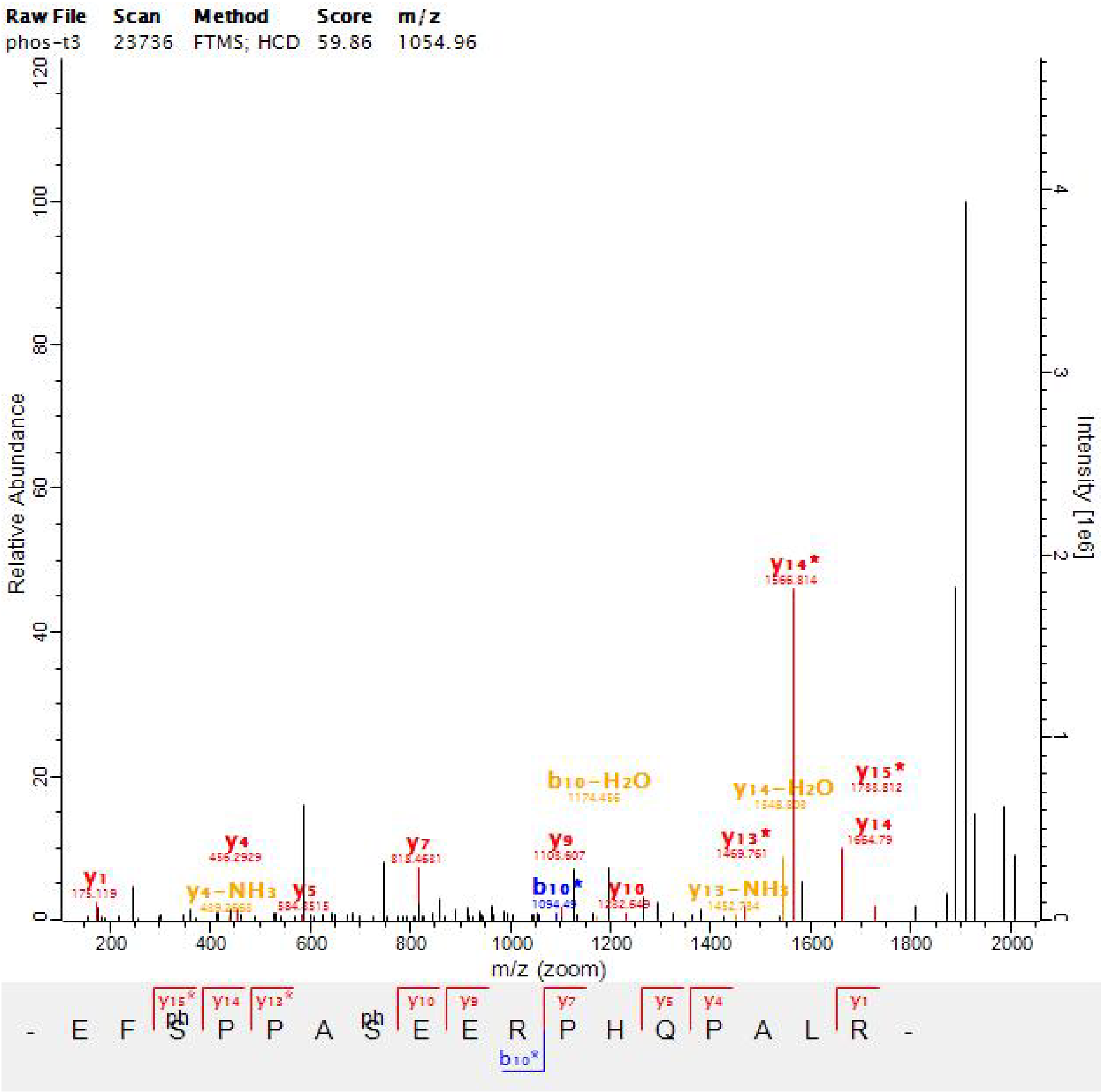

7. Evidence of tf*Ste20*-S517

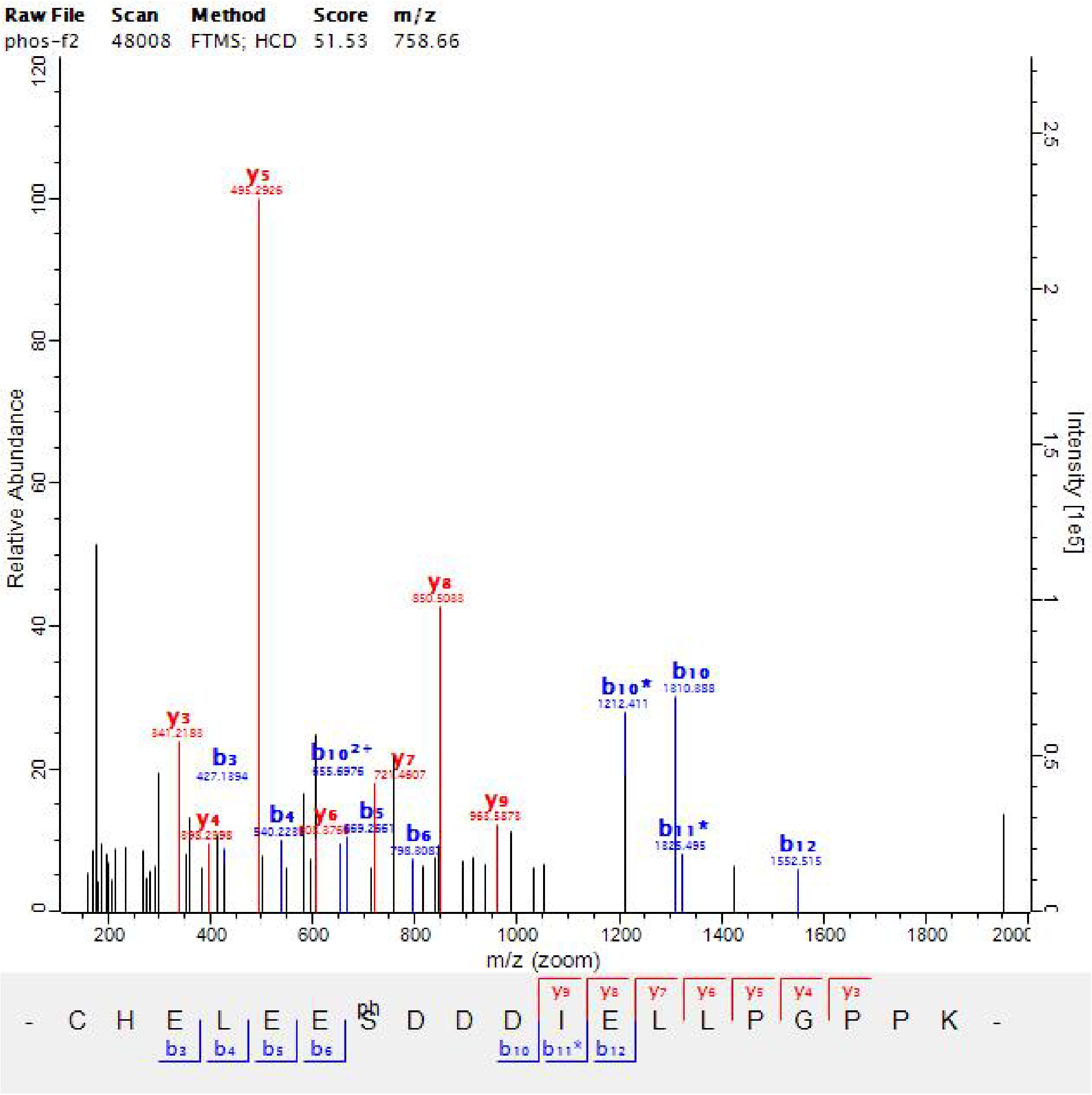

Supplement: Supplementary file 1 [file jof-08-00242-s001.zip › Table S5.pdf]
